# Human genome editing: how to prevent rogue actors

**DOI:** 10.1186/s12910-020-00527-w

**Published:** 2020-10-06

**Authors:** Beverley A. Townsend

**Affiliations:** School of Law, University of KwaZulu-Natal, Durban, South Africa

**Keywords:** Genome editing technologies, CRISPR-Cas9, Ethics and law, Global governance, Harmonisation, Deliberative public engagement, Human rights

## Abstract

**Background:**

Human genome editing technologies offer much potential benefit. However, central to any conversation relating to the application of such technologies are certain ethical, legal, and social difficulties around their application. The recent misuse, or inappropriate use, by certain Chinese actors of the application of genome editing technologies has been, of late, well noted and described. Consequently, caution is expressed by various policy experts, scientists, bioethicists, and members of the public with regard to the appropriate use of human germline genome editing and its possible future effect on future generations.

**Main text:**

As concerns about the applications of heritable genome editing have grown, so too have the questions around what is to be done to curtail ‘rogue actors’. This paper explores various ways in which to regulate genomic editing that are socially beneficial, while being cognisant of legal and ethical principles and rights values. This is done by evolving regulatory frameworks across jurisdictions in an attempt to raise issues, address common principles, and set responsible standards for stewardship of the novel technology.

**Conclusions:**

It is suggested that robust and concrete regulatory measures be introduced that are culturally and contextually sensitive, inclusive, appropriate, and trustworthy – and are based on public empowerment and human rights objectives. Doing so will ensure that we are perfectly positioned to harness and promote the benefits that novel technologies have to offer, while safeguarding public health and curtailing the ambitions of rogue actors. This it is acknowledged is no easy task, so, as a point of departure, this paper sets out a path forward by means of certain, practical recommendations – by constructing genome editing regulation in a manner that both fulfils the desire to better progress human health and that can withstand legal and ethical scrutiny.

The following observations and recommendations are made: Firstly, that a solution of effective, legitimate governance should consist of a combination of national and supranational legislative regulation or ‘hard’ law, in combination with ‘soft’ ethics, firmly anchored in and underpinned by human rights values. Second, that efforts to support legal and ethical solutions should be rigorous, practical, and robust, contribute to a reaffirmation of human rights in a contextually sensitive manner, and be transnational in reach. Lastly, that greater harmonisation across jurisdictions and increased public engagement be sought. This it is proposed will address the question of *how* to implement a normative framework which in turn can prevent future rogue actors.

## Introduction

On 30 December 2019, Chinese biophysicist, He Jiankui, was convicted and sentenced to three years imprisonment and fined 3 million Yuan for the ‘illegal practice of medicine’ by the Shenzhen Nanshan District People’s Court in the People’s Republic of China [1]. This is the consequence of recent experimentation into the realms of human germline genome editing conducted by He, and two colleagues, embryologists, Zhang Renli and Qin Jinzhou. Their efforts caused widespread outrage and consternation within the international scientific community. He and his colleagues illegally conducted genome editing on human embryos intended for human reproduction using the genome editing technique, CRISPR-Cas9. All three pleaded guilty, Zhang and Qin received lesser sentences.

In terms of the judgment, the parties, not qualified to work as doctors, knowingly violated Chinese regulations and ethical principles by practising genome editing in assisted reproductive medicine, by altering embryos in vitro and implanting them into two women [2]. The effect of the edit was to inactivate a gene, the CCR5 gene. Inactivation of CCR5 is associated with HIV resistance, a consequence of which is to hinder the ability of HIV to enter the cells. Twin girls born in November 2018 are the world’s first genetically edited babies – with a third baby expected, but unconfirmed, to have been born in August 2019 [3]. The court found inter alia that the conduct of the parties was a ‘deliberate violation of China’s relevant regulations and medical ethics,’ by ‘the application of human embryonic gene editing technologies for which safety and efficacy have not been proven to clinical practices of assisted reproduction and that their actions went ‘beyond the bottom line of research/clinical ethics’ [4].

He’s actions were widely condemned as medically unnecessary and potentially dangerous to the children – not to mention, sadly, reinforcing the (perhaps, inaccurate) notion that China is an unregulated jurisdiction for research and a playground for speculative and dangerous genetic experimentation. Chinese authorities condemned the practice, announcing at the time He’s work was first made public in November 2018, that the conducted experiments were ‘extremely abominable in nature’ [sic] and in contravention of Chinese law [4]. Significantly, such actions, while infringing upon administrative regulations and ethical norms on genome editing, were not illegal at the time under China’s civil or criminal legislation. The case turned on, and the parties were convicted, solely on the grounds of ‘illegal medical practice’. This is to suggest that had He and his accomplices had medical licences (they ostensibly did not), such medical practices may have been permitted – or at least unregulated. This calls into question the obvious inadequacy of the extant law. Subsequently, and premised on a widespread public outcry and global pressure, the Chinese Civil Code is in the process of being amended to create nuclease germline editing-specific laws [5].

The pressing question, asked both of regulators and the scientific community alike, is how best to stop ‘rogue’ actors in what many may view as dangerous pursuits? While new regulations and ethical rules to govern research and therapies that involve genome editing, gene transfers or attempts to regulate gene expression, and other so-called ‘high-risk’ biotechnologies in humans (or embryos) have been called for, is the behaviour of these actors ‘unethical’ and/or ‘illegal’? What benchmark do we use to hold such actors accountable? Without a normative framework of ethics and law directing their conduct, such activities are neither unlawful nor unethical. While the Chinese authorities have rushed through new laws in an attempt to prevent a similar situation from re-occurring, the question is posed: is this response necessary, reasonable, and sufficient? Chinese proposed new legislation sets in place the same regulations and benchmark standards for germline editing as is required for clinical research on ethically less controversial somatic cells. Certain aspects of the Chinese reaction – rapidly implemented – may not have been carefully considered.

## Genome editing: considerations of *what* and *how*

Although the safety and efficacy of germline editing is yet to be proven, the power to eradicate various diseases and redesign the human genome using genome editing technologies is becoming a certainty. The expectation is that the alteration of the human genome to edit out genetic diseases will morph into the ability to edit ‘normal’ genes, and ultimately to architect selection-specific human genomes [6]. Revolutionising healthcare by editing the genome, and in particular the prospect of using such technologies for non-therapeutic purposes, is not without controversy. Genome editing is predicated on a particular legal, ethical, social and policy construct, the implications and challenges of which, and the desire to re-examine or ‘push’ various ethical and legal boundaries, have been well noted in the literature [7]. Capabilities to edit the genome cannot occur within an ethical and regulatory vacuum. This vacuum can be filled by principles, guidelines, codes, and normative frameworks that guide, direct and govern various aspects of genome editing technologies. Such normative frameworks define *what* standards, guidelines and provisions are required. Once the *what* is established, it is necessary to establish *how* these normative frameworks can be effectively and successfully applied and implemented. This is the process of adopting and implementing the guidelines and provisions into systems, or concrete and workable measures, of practice.

Crucially, however, how these issues and the means of policy adoption will differ between jurisdictions. It is anticipated that, based on public sentiment and opinion, regulations will vary between places as the development of a normative framework follows an approach that is reflective of the moral codes and norms reflective of the wider position held by society in general and the pursuance of a common, greater good [8]. The response to, and adoption of ethical and legal measures differ, and certain countries may thus present a regulatory position where no, or limited, regulation is provided on new technological developments. Thus, disparities and variations exist between the *what* and the *how* of genome editing regulation and oversight in various jurisdictions [9].

This article explores the *what* and the *how* of providing a framework to assist in curtailing rogue actors and proposes various solutions. These include the role of greater harmonisation, increased mechanisms of global governance, engaging the public, and adopting a rights-based approach.

### The role of harmonisation: is it achievable?

Harmonisation is a process by which aspects of legislative, regulatory or policy convergence are identified and differences made compatible [10]. In so doing, common or minimum standards and equivalence across jurisdictions is sought in the nature and adoption of national legislation, regulations, and policies. Harmonisation seeks to effect an approximation or coordination of different legal systems [11].

Harmonisation, collaboration, and cooperation between jurisdictions are notions described in various international instruments. As a point of departure, the Universal Declaration on Bioethics and Human Rights stresses the need to reinforce international cooperation in bioethics – this by taking cognisance of the particular needs of developing countries, indigenous communities, and vulnerable populations [12]. Article 13 stipulates that solidarity among human beings and *international cooperation* towards that end, are to be encouraged. Similarly, the UNESCO Report of the International Bioethics Committee addresses ethical issues associated with the human genome with regard to human rights in ‘Updating Its Reflection on the Human Genome and Human Rights’. The report cautions against unregulated actions resulting from a lack of ethical awareness and ineffective *national and international* regulatory frameworks [13]. An approach of global cooperation and transnational collaboration is also advocated in the U.S. National Academies report [14–16]. A Common Statement issued in 2019 by the Association for the Responsible Research and Innovation in Genome Editing (ARRIGE), the Genome Writers Guild (GWG) and the Japanese Society for Genome Editing (JSGE) called for the launch of ‘a broad and open debate with all stakeholders, including the public’, with regard to the implementation of a comprehensive framework for the international regulation of human genome editing activities [15]. As suggested in a WHO report, what is required is an appreciation of a collaborative, open, inclusive approach based on shared societal values in influencing ethics. One that values diversity and transparency and in turn guides the complexity of genome editing regulation [17]. The WHO has called upon regulators to engage and take action and has called for a central registry for human genome editing research studies, to create an open and transparent database of all ongoing research [18].

A number of governance concerns arise with regard to the application of genome editing to human embryos, the potential and capability for genetic alteration to the human germline, and the access to non-therapeutic genetic enhancements. Such concerns are predicated on the opinions and perspectives of society at a particular time, location and context. As such perspectives on such matters are variable, flexible, and transient in nature – views and opinions of the public are known to change over time and across locations. We can see that opinions and perspectives that at first seem appalling, unnecessary, or incoherent, may and often do soften in time, and perhaps it will be the case with certain attitudes towards genome editing. Moreover, certain jurisdictions may be slower to implement regulatory measures or their law may be silent on matters governing novel technology.

The notion that public acceptance and policy will vary among jurisdictions and lead to divergent policy responses is stated by the Organising Committee of the International Summit on Human genome editing (released on November 2018) [16]. The statement concludes that ‘public acceptability will likely vary among jurisdictions, leading to differing policy responses’ [16].

That being said, engagement and greater cooperation within and between agencies and jurisdictions may be both a worthwhile and a mutually beneficial endeavour. This offers an opportunity to open up collaboration and synergy between international, regional, and local regulators, health administrators, health professionals, academic institutions, and communities. To do so requires fewer prescriptive rules and standards, and an emphasis on increased foundational principles in an overarching governance framework based on human rights. These principles – or core values – should provide a shared form of reference which in turn promotes dialogue and encourages harmonisation between jurisdictions [19]. The clear advantage is that it promotes good practices and builds regulatory capacity [20]. These principles may then form the corpus of what is understood as global health ethics, or an attempt at applying moral value to health issues that are characterised by the need for a global, co-ordinated effort [21].

The proposition is to encourage, to the extent that it is possible, regulatory harmonisation between jurisdictions. Is harmonisation a panacea, though? While a convergence of regulatory approaches would be of benefit, where countries have already adopted diverse laws relevant to a technology, or because the technology is new and countries have no laws whatsoever, harmonisation may not be feasible or likely. The difficulty in getting governance agreement from countries and public consensus should not be understated. Moreover, national responses reflect unique historical, cultural, economic, and social positions which should not be discounted or underestimated. Thus, greater harmonisation, although desirable, may not be easily achievable.

### The role of global governance, ethics and law

How do we begin to regulate disruptive technologies? Particularly, where industry self-regulation and ethical undertakings by scientists to ‘do no harm’ are insufficient mechanisms, in and of themselves, to regulate certain behaviours, and to enforce compliance. The difficulty and urgency is in converting ethical, social and moral reflections on biotechnological problems into practical and workable solutions. Doudna suggests that public conversation and stronger safeguards – based on transparency and accountability – are urgently needed, as ‘moratoria are no longer strong enough as a countermeasure’ to prevent unethical behaviour [22].

There is no international consensus at this time on how human genome editing should be regulated, particularly with regard to the pressing issues of human germline editing and non-therapeutic applications. The first determination is whether ethics instruments will suffice or whether stronger legal-regulatory frameworks are needed. The second is to establish whether any existing ethical and regulatory frameworks sufficiently address the impact of the technologies, and if not, what the gaps are, and how best to address these.

Before attempting to set out a method of governance of genome editing, it is opportune to more carefully describe the relationship between ethics and law in the field of biotechnology, and it particular, human genome editing. Although an oversimplification, ethics is an attempt to answer questions about how, shaped by our values and principles, we *should* conduct our activities, whereas the law creates a basic, enforceable standard of behaviour that we *are compelled* to follow [23]. The law sets out the minimum requirements, whereas ethics typically sets out the maximum requirements. The solution offered by law, albeit clear and concise, may be too limited and rigid in scope, while the failings of ethical solutions are that they all too frequently suffer from conceptual deficiencies and ambiguities, often lacking enforcement mechanisms, remedies and sanction [24]. The relationship between law and ethics is mutually interactive rather than a single flow of influence: ethics influences law in many ways, as law influences ethics [25]. Although ethics and law have developed into separate disciplines, they remain closely connected in their common purpose, that is, the search for appropriate answers to the novel issues facing biotechnology implementation and the compliance with norms shared by law and ethics. Interdisciplinary co-operation between the two disciplines by translating and sharing norms and values may contribute to finding effective solutions – an approach based on a combination of both sound ethical analysis and appropriate legal constructs. The proposal is one of co-operation at the interaction of law and ethics. Law and ethics should complement each other in providing comprehensive governance – therefore regulatory goals should be identified and both legal and ethical responses considered in concert to ascertain the best way to achieve these goals. However, legal and ethical frameworks are often developed in isolation, thus creating something of a patchwork of regulation due in part to the perception of the ‘separate disciplines’ I previously alluded to.

### The role of broader public consultation and deliberative engagement

Increasingly there has been an effort to engage the public in areas of complex and contentious technological endeavours – for instance, by the use of public consultation and engagement to inform biotechnological and environmental policies. Given the unprecedented and persistent interest and public concern over the applications of CRISPR-Cas9, particularly with regard to germline genome editing and enhancement applications, it is necessary to determine, understand and address these concerns by using a process of informed public engagement. The concern is that with the recent birth of the twins in China, interdisciplinary and stakeholder engagement has been replaced by the involvement of expert groupings in an effort to take swift measures to curb unethical genome editing applications of the technology [26].

Public engagement involves educating the public, listening to the public, and empowering the public. Through this mechanism of public discourse, we can navigate the delicate and complex terrain of divergent viewpoints, and the substance and scope of the ethical issues can be more carefully clarified and potentially resolved – this with the ultimate purpose of deriving a ‘broader’ or ‘fuller’ consensus. This approach is endorsed by Jasanoff et al., who state that ‘good governance depends on visions of progress that are collectively defined, drawing on the full richness of the democratic imagination’ [27]. True consensus however may not be readily, or ever, achievable as opinions widely differ, particularly with regard to the far-reaching ethical questions of ‘should genome editing be done’ and ‘under what conditions’. But by opening up the debate and listening to the broader public, the voice of the ‘human community’ can (at least) be heard. A recommendation is to adopt a dual approach – from both the ‘top down’ and the ‘bottom up’.

Ensuring transparency and accountability in the decision-making process, by effective public dialogue and broader stakeholder participation might also increase policy legitimacy. Public inclusion would thus ensure that policy decision-making, at the very least, considers societal views on various contentious issues, which can inform the successful implementation of ethically acceptable and appropriate regulatory policies [28]. Although it may not be possible to obtain consensus, as views and opinions differ, engagement is at least worthy of exploration as it is a approach supportive of greater social cohesion and collaboration, both within societies and between them. These societal discussions and solutions can in turn feed into a global arena, where common issues and values may be debated and shared. Such policy direction should be, however, founded within the parameters of a rights-based framework.

A further, and perhaps more obvious, advantage of heightened public engagement is in securing the public trust in, and acceptance of, new technologies. Trust is a significant determinant in the success of any new technological implementation. Public engagement mechanisms have been applied in various areas of biotechnology to address concerns [29]. Such efforts can go some distance in anticipating the reactions of members of the public to controversial technologies and thus, importantly, to avoid stymying innovation, stigmatising new technologies, or creating barriers to use. Reactions to genome editing applications, for instance, may be elevated by the apprehension over ‘playing God’, the fear of eugenics, or the creation of ‘designer babies’, all views which are deeply influenced by religious, cultural, and historical roots in societies [30–32]. In this way, it is possible to firmly grasp, firstly, what the public’s substantive ethical concerns are with regards to genome editing, secondly, what their attitudes and perceptions are regarding the introduction and application of such technologies into society, and lastly, how their opinions should, and may, be used to resolve these issues and inform policy direction. Public engagement serves to establish a value-based threshold that members of the public find acceptable and which can inform regulatory policy-making.

How much value should be placed on the outcome of public engagement? Public inclusion might ensure that policies take societal views into account [33]. However, it does not follow that because the public agrees on a certain issue or holds a particular opinion, that view is necessarily ethical or legally justifiable. Public opinion should only guide normative frameworks relating to genome editing insofar as they do not infringe upon fundamental human rights – rights which, at times, may need to be carefully balanced.

To obtain democratic legitimacy by following a participatory notion of democracy, policy-makers would not necessarily have to follow what is proposed by groups of the public, but will need to give them due consideration and justify why they select an alternative approach. The purpose of a deliberative process is ultimately to ‘not reflect the position of any particular interest group but rather express a reasoned, informed, judgment forged out of the initially disparate knowledge, values, and preferences of the participants, as these have evolved through the deliberative experience itself’ [34].

Moreover, within this context, can consensus ever realistically be attained? Questions such as whether it is acceptable to genetically engineer changes that will be passed on to future generations, those pertaining to genetic enhancement, and the danger of ‘designing’ offspring according to predetermined, more-highly prized, genetic criteria, flow into particularly tricky terrain. Similarly, questions regarding the best interests of the unborn child, the moral status of embryos, and the prospect of ‘tampering with nature’ may evoke formidable responses. So will divergent views on whether an activity is a ‘line that should not be crossed’ and where that line should be drawn. As opinions are formed within a particular (often deep-seated) philosophical, cultural, religious, and social context, differences are to be anticipated, and even welcomed. But, if by ‘broad societal consensus’ we mean a general agreement on *all* issues by *all* members of society, on certain issues, consensus may well never be reached [35]. Should this then derail the process? I think not. Rather, we should seek to frame the debate in such a way that we speak of an opportunity for the community to engage, to participate, to be heard, and to provide input. The purpose of the engagement is not only to inform and educate, but also to stimulate debate and to be allowed to participate. While in no way diminishing the role and benefit of public engagement, given the plurality of perspectives in society, we should be realistic. We should not lose sight of the aim which is to represent the moral codes and norms reflective of the wider position held by society in general and the pursuance of a common, greater good, not necessarily only the moral indignation of a few or those with clear vested interest [8].

Finally, the ‘public’ is not an undifferentiated mass, so the engagement process should not be designed on the premise that there is a single perspective awaiting identification. Rather, a range of perspectives are likely to exist, ranging from the favourable or compliant to the less so, sparking nuanced perspectives regarding the adoption of genome editing technology and its implications. Fundamentally different views co-exist within most democratic societies, so a plurality of perspectives is to be anticipated and differences in substantive ethical perspectives should not be seen to be problematic [28].

### Towards developing a framework aligned to a rights-based approach

Ethical values are dynamic, and ethical thinking evolves - with various principles gaining increased prominence at different times [36]. Ethical norms and frameworks are expressed to varying degrees within national regulations and determining what is acceptable or permissible, within a context. However, a common thread that runs through most rights-based frameworks is an approach that endorses values and universally accepted human rights: the right to life, the rights of the future child, the right to health care, the right to dignity, the right to equality and non-discrimination, and the freedom of scientific progress. These positions are reflected in, for instance, the Universal Declaration on the Human Genome and Human Rights (UDHGHR) [37], the International Declaration on Human Genetic Data (IDHGD) [38], and the Universal Declaration on Bioethics and Human Rights (UDBHR) [39].

In considering the application of genome editing technologies and research various rights come into play. Freedom of scientific researchers is one such right, and should be balanced carefully with due regard to other rights [13]. One central and critical focus should be on the equitable distribution of, and access to, expensive technologies and the scalability of therapeutic applications – that is, issues of equitable access to, and participation in, social justice and to such technologies [40].

Where governance frameworks are inappropriate or incomplete, this not only creates barriers to scientific and clinical advancements, but compromises individual rights protection. Thus, the introduction of an international human rights-based framework will address ethical issues, and serve as a valuable point of departure from which to launch practical solutions within the context of regulating novel technologies [41]. The principles contained within these frameworks might then be replicated in regional, national, and supranational legislative structures. As suggested by Brokowski and Adli, the development of such national and supranational regulatory measures, although unlikely to eliminate all risk, may reasonably manage and minimise it [42]. The WHO may be best positioned to lead the way in this regard and create such a guidance framework [43]. To this end, the WHO has established a committee with the purpose of examining the scientific, ethical, social, and legal challenges associated with human genome editing, establishing global standards for the governance and oversight thereof, and advising and making recommendations on appropriate governance mechanisms [44]. An analysis, review and amendment of existing international, national and regional regulations may be needed [40]. Particular consideration is needed primarily of the value of scientific progress, scientific benefit, benefit sharing, the equitable access to new therapies, and the implication and role of human germline therapies and enhancements to society – with a focus on the underlying principles of transparency, inclusiveness, responsible stewardship of science, fairness and social justice. Much may be achieved by leveraging off of existing international frameworks. In addition, international organisations, such as ARRIGE, could be instrumental in providing support and guidance in establishing a path forward. Such approaches by non-governmental organisations are beneficial in encouraging a forum of transnational shared dialogue and networking on issues of regulation and governance.

Worthy of mention is the call for the establishment of a global observatory for genome editing consisting of an international network of scholars and organisations dedicated to collecting data from dispersed sources – thereby bringing together previously disregarded perspectives, and encouraging the sharing of ideas and co-operation across disciplinary and cultural divides [45]. This is a view proposed by Jasanoff and Hurlbut and entails a coordinated international effort of integrating perspectives from science and society, with the aim of uncovering divergent ideas and making suggestions on how best to protect human rights with respect to biotechnological advancements [45]. This process should encourage sustained international and interdisciplinary reflection on several key considerations, including: the questions to be asked, whose views should be heard, what imbalances of power should be made visible, and what diversity of views exist globally [45]. Divergent perspectives should be represented, and existing approaches should be recalibrated in the light of alternative positions [45], thereby introducing a position of greater global integration and consolidation.

## Managing risk in the uncertain world of genome editing and exercising caution

Ethics is founded in norms and values – and describes the way society ‘ought to be’ or ‘should be’ [46]. At times, it is not clear how much benefit or harm is being done. Genetic therapeutic interventions occur within a world where absolute certainty can never be realistically achieved, and that is why it is by definition ‘uncertain’ and therefore intrinsically unknowable. Risk on the other any hand may be reasonably determined based on probabilistic outcomes and might be measured, quantified, and managed. To properly manage risk, however, we need to understand the probabilities we are dealing with, including the risk of very unlikely and very bad events occurring. Whether we are prepared to assume risk will depend, thus, on how well we understand the possible outcomes and on our capacity to be able to successfully manage the risk posed by genome editing. Essentially, it amounts to an act of carefully balancing the probabilistic risks with the potential rewards.

This does indicate a need for caution – as, like Icarus, we too may be burned by the sun, if hubristically, we ignore the possibility that things could, and do, go wrong and not take measures to prevent harm to humankind, the environment, and our future. But caution should not be used as an excuse to avoid us *understanding and appreciating* the risks and taking steps to *manage* them. It is only then that we can be expected to make informed decisions. Given the balance of risks and benefits, what these decisions may require of us is determining, not what is safe, but what is ‘safe enough’ and what this means within the context of genome editing. Where the potential for harm is increased the technology would require a more restrictive regulatory framework.

With regard to a determination of biosafety, much turns on the presumption about risk – either one of precaution (presuming danger until safety is proven) or one of permission (presuming safe until the contrary is proven) [47]. Certain benchmarks will need to be set, but how do we determine these benchmarks and who is to make the final determination?

Benefits may well outweigh the risks in certain circumstances. Given the promise of this technology to relieve human suffering and support human flourishing, there may be a moral imperative in certain cases to proceed along this path. In an uncertain future, how can we be certain that the inactions of today will not worsen the future position, rather than ameliorate it? By doing nothing, we may be in effect denying others valuable treatment options. So, although genome editing may carry plausible risk, failing to pursue such technology may likewise carry resultant and equally plausible outcomes. We should not, however, forgo the worthy pursuits of addressing shared human needs, societal health priorities, therapeutic development (particularly in cases of dire conditions), and the value of autonomy (by not denying patient treatment options) without a clear path forward.

The suggestion is that genome editing be approached in a way that is sensible and responsible, and regulated – this means evidence-based determinations and the assessment of probabilistic predictions and outcomes, and crucially, the correct checks and balances must be put in place and strictly adhered to. The social and ethical implications of adopting these technologies should be carefully considered and clear parameters set to enable actors to understand the normative framework within which they may (or may not) lawfully and ethically operate. What is clear, however, is that we cannot allow rogue actors to make these irreversible decisions for us all.

## Summation: A way forward

Four distinct observations and recommendations are made. Firstly, the suggestion is that a solution of effective, legitimate governance should consist of a combination of national and supranational legislative regulation or ‘hard’ law, in combination with ‘soft’ ethics, firmly anchored in and underpinned by human rights values. Neither an approach solely reliant on ‘soft law’ nor on industry self-regulation will be sufficient – as inclusive, democratic legitimacy is needed [26]. While ethics and ethical guidelines are of value and can make a significant contribution to resolving the various dilemmas, such ethical guidelines should not be used as a placeholder for robust, appropriate legal frameworks, but should be complementary to, rather than a substitute for, the law [48].

Secondly, in proposing a normative framework, it is suggested that any guidance must: (1) be sensitive to societal and cultural differences in what is considered appropriate and realistic, (2) be responsive, proactive and flexible to the rapid technological transformation in the biotechnology industry, (3) foster and build confidence of the public in providing adequate protection measures within this context, and (4) align with global rights-based best practice, principles and standards. This will encourage and enable implementation and facilitate much needed confidence and trust in the new technology – an approach therefore that provides a regulatory baseline comprising clearly defined, yet contextually and culturally sensitive principles, that is cognisant of differing societal needs and which endorses democratic participation. In this way, regulation and governance is underpinned by rights principles, and is based on considerations of consensus that take socioeconomic issues into account. The solution is in building a comprehensive institutional system to effectively guide, manage, regulate and then enforce biotechnology policy.

Thirdly, a global instrument to assist in establishing a stable list of non-arbitrary standards and principles that justifies and endorses certain values, ethics and rights should be developed. This would assist in issues of global governance and provide a much needed regulatory framework. It should be borne in mind that *informal* mechanisms of international cooperation do not impose legal requirements on the individual countries or states to implement specific provisions, but rather provide general agreement between countries in the form of non-binding guidelines to do so.

The adoption of *formal* international instruments or conventions, however, requires ratification by member countries and then incorporation into national or domestic legislation. Although they have normative value, their enforcement-level is often inconsistent, in that state practice determines what eventually becomes settled as norms, values and rules [49]. In the case of most conventions, they are not self-executing instruments. A ‘self-executing treaty’ is enforced directly into national law without enactment by domestic legislation. Most international instruments or conventions would offer a malleable template or guide for advancing legislation, which can be modified by member states to fit their national peculiarities and requirements [50]. Countries which accede to and ratify the treaty, however, would commit to establishing a legal regime based on its provisions and the obligations created therein may provide a useful catalyst for the evolution of legal frameworks around the world. Although harmonisation is not entirely achievable, notwithstanding these difficulties, there is compelling value in an approach of coordination and collaboration between regulators in different countries, where feasible. Not least to provide opportunities for identifying common ground on specific substantive or technical aspects and in creating a valuable framework for adoption [51].

Lastly, in finding a solution we should not lose sight of the tremendous potential benefit genome editing affords – particularly, somatic cell editing. As the promise of germline editing is still under question (as noted in the literature) how useful it might be and its safety and efficacy is still to be established. We have an entire toolbox of instruments, guidelines, measures and platforms at our disposal from which to proactively develop ethical and legal safeguards so that technology can be allowed to progress for the benefit of humankind.

## Conclusion

In the final analysis, much can be done to curtail rogue actors. Genome editing technologies are often referenced as key developments in future therapeutic and non-therapeutic applications. Central to any conversation relating to the application of such technologies are certain ethical, legal, and social difficulties around their application. Potential forces of ethical and unlawful misuse of new technologies increase, unless their existence is checked and actors are held accountable. Although the response from the public and scientific community assisted, interestingly, and perhaps tellingly, it was the Chinese courts and national law that ultimately held the actors accountable and provided a remedy.

It is only when an actor moves beyond the limits of what is deemed lawful and ethical (and by implication reasonable), that they can be called a ‘rogue actor’. Having laws within a sovereign state, and enforcing those laws, is the best way to stop rogue actors and to prevent such behaviour from re-occurring.

Discussions and cooperative efforts at a global level should continue to review and keep abreast of the evolving regulatory frameworks across jurisdictions in an attempt to raise issues, address common principles, and set responsible standards for stewardship of the technology. This allows for proactive rather than reactive decision-making and can provide for an ethical and regulatory toolbox of formal and informal rules, guidelines, protocols and practices for appropriate genome editing governance. Additionally, cooperative efforts help determine the acceptable ethical boundaries of genome editing and should be a priority, so that the diversity of societal, cultural, and political values become an asset rather than a hindrance in reaching consensus [17]. This is a perspective supportive of an ‘ecosystem’ approach to the regulation of novel biotechnologies. It serves to take ‘advantage of the ecosystem of regulatory actors and develop a road map for responsible translational research’ that includes ‘stringent criteria for use of germline editing and standards for determining whether these criteria have been met, embedded within larger political structures offering vehicles for public input’ [52].

Regulatory measures may be introduced that are culturally and contextually sensitive, inclusive, responsive and trustworthy – and are based on public empowerment and human rights objectives. This being said, while many approaches may provide partial or incomplete answers, an integrated, holistic solution to global enforcement and remedy is not easily done, but the above recommendations may go some way in providing a path forward.

We are at an interesting juncture in the discourse on the regulation of genome editing technology. Efforts to support legal and ethical solutions should contribute to a reaffirmation of human rights in a contextually sensitive manner, and be transnational in reach. It is suggested that this necessitates greater harmonisation across jurisdictions, increased public engagement, a framework endorsing a rights-based approach, and a multi-layered regulatory approach consisting of both ‘hard’ and ‘soft’ law. This it is proposed will address not only the question of *what* but also of *how* to implement a normative framework, which, in turn, can prevent future rogue actors.

## Data Availability

Not applicable.
